# Analysis of the Link between the Redox State and Enzymatic Activity of the HtrA (DegP) Protein from *Escherichia coli*

**DOI:** 10.1371/journal.pone.0117413

**Published:** 2015-02-24

**Authors:** Tomasz Koper, Agnieszka Polit, Anna Sobiecka-Szkatula, Katarzyna Wegrzyn, Andrea Scire, Donata Figaj, Leszek Kadzinski, Urszula Zarzecka, Dorota Zurawa-Janicka, Bogdan Banecki, Adam Lesner, Fabio Tanfani, Barbara Lipinska, Joanna Skorko-Glonek

**Affiliations:** 1 Department of Biochemistry, Faculty of Biology, University of Gdansk, Gdansk, Poland; 2 Department of Physical Biochemistry, Faculty of Biochemistry, Biophysics, and Biotechnology, Jagiellonian University, Krakow, Poland; 3 Department of Molecular and Cellular Biology, Intercollegiate Faculty of Biotechnology of the University of Gdansk and Medical University of Gdansk, Gdansk, Poland; 4 Department of Life and Environmental Sciences, Universita Politecnica delle Marche, Ancona, Italy; 5 Department of Biochemistry, Faculty of Chemistry, University of Gdansk, Gdansk, Poland

## Abstract

Bacterial HtrAs are proteases engaged in extracytoplasmic activities during stressful conditions and pathogenesis. A model prokaryotic HtrA (HtrA/DegP from *Escherichia coli*) requires activation to cleave its substrates efficiently. In the inactive state of the enzyme, one of the regulatory loops, termed LA, forms inhibitory contacts in the area of the active center. Reduction of the disulfide bond located in the middle of LA stimulates HtrA activity *in vivo* suggesting that this S-S bond may play a regulatory role, although the mechanism of this stimulation is not known. Here, we show that HtrA lacking an S-S bridge cleaved a model peptide substrate more efficiently and exhibited a higher affinity for a protein substrate. An LA loop lacking the disulfide was more exposed to the solvent; hence, at least some of the interactions involving this loop must have been disturbed. The protein without S-S bonds demonstrated lower thermal stability and was more easily converted to a dodecameric active oligomeric form. Thus, the lack of the disulfide within LA affected the stability and the overall structure of the HtrA molecule. In this study, we have also demonstrated that *in vitro* human thioredoxin 1 is able to reduce HtrA; thus, reduction of HtrA can be performed enzymatically.

## Introduction

Proteins targeted to the cell envelope in Gram-negative bacteria reach the periplasmic space in an unfolded state and require subsequent folding [1]. In contrast to the cytoplasmic environment, the environment of the periplasmic space is oxidizing and formation of disulfide bridges in proteins is favoured [2] [3]. Formation of the appropriate S-S bonds is crucial for the proper folding of numerous proteins in the envelope, as they stabilize their tertiary and/or quaternary structure [4]. Failure to form proper disulfide bridges, or a too slow oxidation of cysteines, may lead to aggregation of proteins or their degradation by proteases [5] [6]. Spontaneous disulfide bond creation is very slow and, thus, this process is catalyzed by a specialized group of oxido-reductases. In the periplasm of *Escherichia coli*, the S-S bonds are introduced by Dsb proteins—DsbA and DsbB—which are responsible for sulfhydryl group oxidation and DsbC/DsbG, and DsbD, which are involved in the isomerization of wrongly introduced disulfide bonds [7].

Improperly folded proteins (e.g. those lacking proper disulfide bonds) represent a potential threat for a cell and must therefore be removed [8] [9]. In the periplasm, such a function is fulfilled by an HtrA (DegP) serine protease/chaperone which participates in the degradation or folding of damaged extracytoplasmic proteins, as well as in keeping the unfolded proteins in a soluble state [10].

In a model Gram-negative bacterium, *Escherichia coli*, HtrA is a peripheral inner membrane serine protease which is indispensable for the survival of cells at temperatures above 42°C and in the presence of certain oxidizing agents. Its synthesis is induced under various stress conditions, including heat shock, oxidative stress and the presence of reducing agents [11] [12] [13] (reviewed in [14]). The proteolytic activity of HtrA strongly depends on temperature, with the highest activity observed at temperatures above 45°C [15]. At lower temperatures (below 28°C), this activity is hardly detectable [16]. However, under conditions that disturb formation of disulfide bonds HtrA efficiently degrades its natural substrate, reduced alkaline phosphatase, even at temperatures as low as 20°C both *in vivo* and *in vitro*. [17]. This ability was especially pronounced in the case of the cysteine-less HtrA variant which lacked its intramolecular S-S bond (ΔCys HtrA). Thus, we concluded that the reduction of the HtrA disulfide bridge may facilitate the activation of the protease. HtrA, in its native conformation, contains a disulfide bond which is formed by cysteines 57 and 69 and is introduced by DsbA oxidase [17] [18]. However, under reducing conditions or in the absence of DsbA, HtrA is present mainly in a reduced state in the cell [18] [19].

Cys57 and Cys 69 are parts of the LA loop (residues 38–79), which is one of the regulatory loops of the HtrA molecule. The proteolytic domain of HtrA shows a structural scheme that is typical of the chymotrypsin type of proteases. This is composed of two β-barrels, whose secondary elements are connected by loops, including the LA, LD, L1, L2, and L3 regulatory loops. HtrA is a protein which requires activation to exhibit its proteolytic activity. The protease active site triad (comprising residues His105, Asp135, and Ser210), is placed at the interface of the β-barrels and in the resting state of the enzyme is not properly organized and is inaccessible for substrates. The inactive HtrA is a hexameric molecule composed of two trimeric units connected by C-terminal PDZ domains and additionally by long LA loops that protrude into the opposite trimer. The LA loop is believed to play an inhibitory role by stabilizing the inactive conformation and shielding the active center. Specifically, the LA of the opposite subunit (LA’) interacts with L1, the loop which contains an active site Ser210, and with the L2 regulatory loop which is responsible for substrate binding [20] [21]. In particular, the Asn45, Gln47 and Gln70 of the LA’ loop interact with the Asn206, Arg207 and Asn209 of the L1 loop. The hydrophobic residues of the L2 loop (Ile228, Leu229, Ile236) are in contact with Cys 69, Phe56 or Phe68 of the LA’ loop. Moreover, those LA’ parts protruding into the opposite trimer form contacts with each other by means of hydrophobic residues (Phe and Pro residues at positions 46, 49, 50, 62, 63, 67, and 68) and stabilize the inactive conformation of HtrA [22] [23]. We have recently demonstrated that the majority of the mutations within the LA loop which destabilize the interactions listed above lead to increased activity of HtrA, especially at low temperatures [23]. The activation process requires significant structural rearrangements and it can be triggered by a temperature shift or allosterically by peptide binding. During thermal activation, the LA loop was the first element to respond to a change in temperature. The degree of exposition of LA to the solvent positively correlated with the increase in the temperature and stimulation of the proteolytic activity of HtrA [24]. Allosteric activation involves the attachment of an activator, a peptide capable of binding to both the active center and to the C-terminal PDZ1 domain simultaneously. The binding triggers a cascade of structural changes involving the transmission of a signal by the regulatory loops. As a result, the active center becomes released from the LA’ loop and adopts a catalytically competent conformation. This process is accompanied by the reorganization of the oligomeric structure: the hexamer of HtrA dissociates transiently into trimers and subsequently forms higher-order oligomers [20] [21] (reviewed in [25]). Certain mutations within the LA loop also affect the assembly of HtrA monomers; in most cases, a destabilization of the resting state hexamers is observed [23] [20].

The proteolytic activity of HtrA has been reported to be important for the virulence of several pathogenic bacteria [14] [26]. In uropathogenic *E*. *coli* strain CFT073, the proteolytic activity of HtrA is necessary for the virulence in a mouse model of urinary tract infection [27]. In the pathogenesis of *Salmonella* HtrA, proteolytic activity is important for efficient amplification in the liver and spleen of mice during infection [28]. Particularly interesting are species which can secrete HtrA into the extracellular milieu. To this group belong enteropathogenic Gram-negative bacteria (*Helicobacter pylori*, *Campylobacter jejuni*, *Shigella flexneri*, and enteropathogenic *E*. *coli* strains) whose HtrA participates in the disruption of epithelial layers. In particular, HtrA degrades E-cadherin, a component of adherence junctions. In the case of the best studied example, *H*. *pylori*, inhibition of the activity of extracellular HtrA strongly limits invasion of the host. Secreted HtrA of *C*. *jejuni* is also capable of degrading E-cadherin and its absence leads to a strong defect in the adherence to host cells and cellular invasion [29]. Therefore, it has been postulated that cleavage of E-cadherin mediated by HtrA is an important mechanism in the pathogenesis of Gram-negative gastrointestinal bacteria. The significance of HtrA in bacterial virulence and survival under stressful conditions makes this protein an interesting therapeutic target. Development of specific and efficient anti-HtrA molecules requires a thorough characterization of HtrA’s mode of action and its regulation. Reversible reduction of the Cys57-Cys69 disulfide bond seems to be an additional opportunity to tune the proteolytic activity of HtrA. To prove this hypothesis, we performed a detailed biochemical and biophysical characterization of the reduced and cysteine-less variants of HtrA. We found that HtrA deprived of its S-S bond was characterized by a higher turnover of a model peptide substrate, bound a model protein substrate more efficiently, and was converted to a higher-order-oligomeric form more easily when compared to an HtrA version containing its disulfide bond. In this study, we also discuss the potential physiological importance of HtrA reduction. We demonstrate that HtrA can be a substrate for human thioredoxin 1; thus, it is possible that exported fractions of HtrA become enzymatically reduced in the intestines.

## Materials and Methods

### Materials

Deuterium oxide (99% ^2^H_2_O) and ^2^HCl were purchased from Aldrich. DNA polymerase Pfu Ultra Hotstart was purchased from Agilent Technologies (Santa Clara, CA, USA); bis[sulfosuccinimidyl] suberate (BS^3^) was from Pierce Biotechnology (Rockford, IL, USA). The primers used in site-directed mutagenesis were purchased from Proligo (Boulder, CO, USA). Other chemicals were purchased from Sigma-Aldrich (St. Louis, MO, USA) and were of the highest purity. The NWVSAA↓KFE-Y^NO^_2_-O2Oc-O2Oc-IYQV (PepPEG) and NWVSAAKFESTDGSTDYGIYQV (22-peptide) were synthesized on solid phase using Fmoc/tBu strategy as it was described in [30] and [31], respectively.

### Strains and plasmids

The strains and plasmids used in this work are listed in Table 1.

**Table 1:** Bacterial strains and plasmids.

strain/plasmid	genotype	reference
*E*. *coli* K38	HfrC(λ)	[32]
*E*. *coli* BL20	W3110 *htrA63 galE sup* ^+^	[33]
pGP1–2	pACYC177, *1*(T7 RNA polymerase) cI857	[34]
pJS18	pQE60, wt *htrA* with C-terminal 6×His tag	[19]
pJS17	pQE60, *htrA*-S210A with C-terminal 6×His tag	[17]
pAMS3	pQE60, *htrA*-C57A/C69A with C-terminal 6×His tag	[17]
pAMS18	pQE60, *htrA*-C57A/C69A/S210A with C-terminal 6×His tag	[17]
pJS14	pT7–5, *htrA-*S210A	[35]
pJS27	pT7–5, *htrA-*C57A/C69A/S210A	this work
	BamHI-BstEII segment of pJS14 substituted with a pAMS18 fragment containing the C57A/C69A mutation	
pW214/pAW63	pT7–5, *htrA-*F63W/S210A	[24]
pTK1	pT7–5, *htrA-*F63W/C57A/C69A/S210A	this work
	Site-directed mutagenesis with F63W and F63W-RW primers (QuikChange II protocol) on a pJS27 template. F63W: 5’-GGAAGGTTCTCCGTGGCAGAGCTCTCCGTTCG-3’; F63W-RW: 5’-CGAACGGAGAGCTCTGCCACGGAGAACCTTCC-3’	

### Protein purification

HtrA variants without C-terminal 6×His tags were overproduced in *E*. *coli* K38/pGP1–2 strain transformed with the appropriate plasmid in the T7 promoter/polymerase system [34], and these variants were purified as described previously [18]. HtrA variants with C-terminal 6×His tags were overproduced in the BL20 strain transformed with the appropriate plasmid in the T5 promoter system and were purified as described previously [17]. The purity of the obtained protein preparations was estimated to be approximately >95%, as determined via sodium dodecyl sulfate polyacrylamide gel electrophoresis (SDS-PAGE). The concentration of the preparations was estimated using amido black staining, as described previously [36].

### HtrA protease assay

Kinetics of proteolysis catalyzed by HtrA was carried out using the substrate NWVSAA↓KFE-Y^NO^_2_-O2Oc-O2Oc-IYQV (PepPEG), a derivative of the p23 fluorescence substrate described in [37]. The hydrolysis of the peptide bond occurs between the alanine and lysine residues and is marked with an arrow. A Trp indole ring served as a fluorescent probe, 3-nitrotyrosine (Y^NO^_2_) served as a fluorescence quencher. O2Oc stands for 8-amino-3,6-dioxa-octanoic acid (a functionalized linker equivalent in length of 3 amino acid residues). PepPEG was dissolved in dimethyl sulfoxide (99.5% DMSO), then diluted by a factor of 10 in 20 mM HEPES-NaOH pH 8.0, 100 mM NaCl and stored as stock solution in -80°C.

Various concentrations of PepPEG were pre-incubated for 5 minutes at 20°C in 20 mM HEPES-NaOH, pH 8.0, 100 mM NaCl, then HtrA variants with C-terminal 6×His tags were added to final concentrations of 0.1 mM and the initial rates of increase in fluorescence intensity were recorded. The measurements were carried out using a Perkin Elmer LS55 Fluorescence Spectrometer in 1-mm path length cells. The excitation and emission wavelengths were 282 and 360 nm, respectively, and 10 nm bandpasses were used for both excitation and emission.

The level of hydrolysed PepPEG was calculated from a calibration curve of the fluorescence intensity of 1:1 (molar) mixture of NWVSAA and KFE-Y^NO^_2_-O2Oc-O2Oc-IYQV. The empiric data from three independent measurements were then fitted to the Hill form of the Michaelis-Menten equation *v*_0_ = *V*_max_ × [S]^*n*^ × (*K*’ + [S]^*n*^)^-1^, where *v*_0_ is the observed initial cleavage rate, *V*_max_ is the apparent maximum cleavage rate, *K*’ is the apparent Michaelis constant, and *n* is the Hill coefficient [37]. The fitting was performed using Origin software (Northampton, MA, USA).

### Surface Plasmon Resonance (SPR)

Standard SPR analysis was performed on a BIAcore 2000. β-casein binding by the HtrA variants (100, 150, 200, 250, 300 nM) was studied using β-casein immobilized on a Sensor Chip CM7 (GE Healthcare). The running buffer was HBS-EP (150 mM NaCl, 10 mM HEPES pH 7.4, 3 mM EDTA, 0.005% Surfactant P20). In all the experiments the buffer flow was set to 15 μl/min, with all the injections at a volume of 30 μl. The final sensorgrams were obtained after subtraction of the background response signal from control experiments with buffer injections.

### Spectral Measurements

For infrared measurements, about 1.5 mg of the purified HtrA protein was transferred into a 30 K Centricon micro concentrator (Millipore), centrifuged at 3000 × g at 4°C and concentrated into a volume of approximately 40 μl. Then, 300 μl of 20 mM Tris-^2^HCl, 20 mM KCl p^2^H 8.0 (buffer A) or 20 mM Tris-^2^HCl, 20 mM KCl, 1.5 mM dithiothreitol (DTT) p^2^H 8.0 (buffer B) were added and the samples were concentrated again. The p^2^H value corresponds to the pH meter reading + 0.4 [38]. The concentration and dilution procedure was repeated several times in order to completely replace the original buffer with buffer (A) or buffer (B). The washings took 24 hours, that is the contact time of the protein with the ^2^H_2_O medium prior to Fourier Transform Infrared (FT-IR) analysis. In the last washing, the protein sample was concentrated to a final volume of approximately 40 μl and used for the infrared measurements. The spectra of protein samples were collected and processed as described in [24]. The protein melting temperature (T_m_) was calculated by fitting the values of the second derivative band intensities at 1634 cm^-1^ or 1617 cm^-1^ to the sigmoidal Boltzmann curve using Origin software (Northampton, MA, USA).

The near-UV circular dichroism (CD) spectra (250–330 nm) of HtrA variants (89–182 μM) were recorded in 20 mM Tris-HCl pH 8.0, at a temperature range of 20–45°C (5°C steps) in a 1-mm path length cell using a JASCO J-815 (Japan) spectropolarimeter. Each spectrum represents an average of three accumulations of protein pre-incubated at a given temperature for 5 minutes. The spectra of buffer were subtracted from the protein spectra. The mean residue ellipticity [*θ*]_mr,λ_ at a given wavelength λ was calculated using the equation [*θ*]_mr,λ_ = *θ*_λ_ × *M* × (*N*—1)^-1^ × (10 × *c* × *d*)^-1^, where *θ*_λ_ is the observed ellipticity (in degrees) at wavelength λ, *M* is the molecular mass of HtrA (in daltons), *N* is the number of amino acid residues in the HtrA polypeptide chain (448 residues), *c* is the protein sample concentration (in g/ml), and *d* is the cuvette pathlength (in cm) [39].

Steady-state fluorescence spectra, fluorescence quenching and time-resolved fluorescence decay measurements were carried out according to [24].

### Reversed-phase high performance liquid chromatography (RP-HPLC)

100 μl samples of reaction mixtures containing 20 μM HtrA variants were acidified by the addition of formic acid to a final concentration of 9% and applied to a Zorbax 300SB-C8 (Agilent, 3 × 150 mm, 3.5 μm) column equilibrated with 30% acetonitrile (ACN) and 0.1% trifluoroacetic acid (TFA). The samples were eluted with a linear gradient of 30–55% ACN in the presence of 0.1% TFA at a flow rate 0.25 ml/min.

### Protein cross-linking and size-exclusion chromatography (SEC)

The HtrA variants (10 μM monomer) were preincubated for 15 minutes at 37°C with or without a 22-peptide ligand in 20 mM HEPES-NaOH pH 8.0, 100 mM NaCl, and then incubated for 10 hours at 4°C. Then, the samples were treated with the addition of BS^3^ to a final concentration of 0.45 mM and incubated at room temperature for 30 minutes. The cross-linking reaction was terminated by the addition of Tris-HCl buffer (pH 7.5) to a final concentration of 50 mM.

SEC was carried out on a Superose 12 10/300 GL column (GE Healthcare) equilibrated with 50 mM Tris-HCl, pH 8.0, 100 mM NaCl. 50 μl samples of the cross-linked HtrA species were analyzed at 25°C at a flow rate of 0.3 ml/min.

The elution volumes of molecular weight standards (Bio-Rad) were used for column calibration and calculation of the HtrA monomer level in each eluted cross-linked species.

### Enzymatic reduction of HtrA by human thioredoxin 1 (Trx1)

HtrA-S210A (20 μM) was incubated with 0–2.0 μM Trx1 in a buffer containing 20 mM KHPO_4_ pH 7.5, 2 mM DTT, 2 mM EDTA (optionally with the 22-peptide ligand) for 15 minutes at 37°C. The reaction was stopped by the addition of formic acid to a final concentration of 9% and the oxidized and reduced forms of HtrA were resolved via RP-HPLC, as described above.

## Results

### Cysteine-less HtrA cleaves a peptide substrate more efficiently than wt HtrA

To test the importance of the Cys57-Cys69 disulfide bridge for the proteolytic activity of HtrA, we compared the kinetics of single peptide bond cleavage by ΔCysHtrA and wt HtrA using a synthetic substrate PepPEG. This substrate contains the N-terminal single cleavage-site degron and the C-terminal PDZ1-binding degron. Both degrons are connected with a PEG-derived linker of a length corresponding to 6-amino-acid peptide. PepPEG proved to be efficiently cleaved by HtrA. This finding supported the hypothesis that the particular sequence linking both degrons is not important, as long as it provides a covalent connection of an appropriate length. The electron density for the peptide linker region between the PDZ1-binding and the cleavage-site degrons was not traced by crystallography [40]; thus, most probably, the linker’s residues do not form stable interactions with the HtrA molecule.

**Fig 1:**
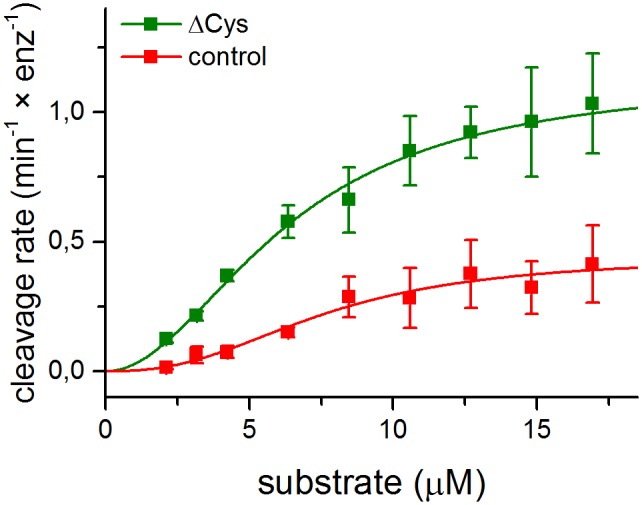
Kinetics of proteolysis catalyzed by HtrA. Initial rates of ΔCys (HtrA-C57A/C69A-6×His tag) and oxidized control (wt HtrA-6×His tag) variant (0.1 μM monomer) cleavage of different concentrations of PepPEG (NWVSAA↓KFE- Y^NO^_2_-O2Oc-O2Oc-IYQV) were measured at 20°C, as described in “Materials and Methods”. The empirical data were plotted against the Hill equation. The error bars represent the standard deviation values from three independent measurements.

We titrated PepPEG against HtrA and assayed its cleavage rates (Fig. 1). The fluorescent substrate cleavage followed sigmoidal kinetics characteristic for allosteric enzymes, with the parameters provided in Table 2. As can be seen, the *K*’ values of both HtrA variants were comparable, but the *V*_max_ value of ΔCysHtrA at 20°C was more than twice as high as that of wt HtrA and comparable to the *V*_max_ exhibited by wt HtrA at 37°C (0.95 ± 0.03 min^-1^ × enz^-1^). At 37°C the difference was less pronounced; however, the turnover of the substrate by ΔCysHtrA was still approximately 1.5 fold more efficient (1.56 ± 0.03 min^-1^ × enz^-1^). Hence, the lack of the S-S bond leads to the stimulation of the proteolytic activity of HtrA, especially at low temperatures.

**Table 2:** Kinetic values of proteolysis catalysed by HtrA.

	ΔCys (C57A/C69A)	oxidized control
*V*_max_ (min^-1^ × enz^-1^)	1.13 ± 0.04	0.44 ± 0.03
*K’* (μM)	6.38 ± 0.31	7.59 ± 0.65
n	1.99 ± 0.15	2.52 ± 0.43

*V*_max_—apparent maximum cleavage rate, *K*’—apparent Michaelis constant, *n*—Hill coefficient.

### The lack of a Cys57-Cys69 S-S bond increases the affinity of HtrA to a protein substrate

**Fig 2:**
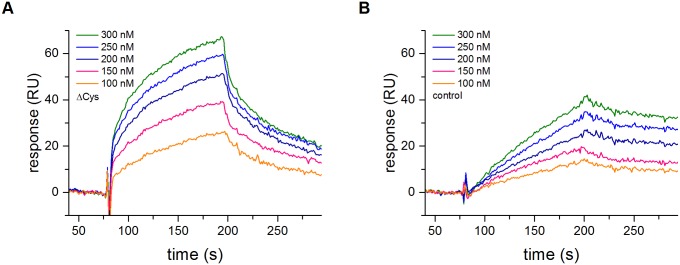
Sensorgrams of HtrA affinity to β-casein. (A) affinity of ΔCys (HtrA-C57A/C69A/S210A–6×His tag) variant. (B) affinity of oxidized control (HtrA-S210A–6×His tag) variant. Increasing amounts (100–300 nM) of HtrA variants were run over the surface of a sensor chip with immobilized β-casein, as described in “Materials and Methods”. A representative sensorgram is shown; RU, response units.

Similar values for *K*’ constants suggested that both HtrA versions exhibit a similar affinity to the tested peptide substrate. However, the natural substrates for HtrA are denatured or misfolded proteins rather than peptides. Association with such a large substrate is mediated by much larger surfaces than those of the active center and the peptide binding groove in PDZ1, at least at the initial step. Thus, the affinity towards a protein substrate may differ from that towards a peptide substrate. To check this, we performed an SPR analysis of both HtrA variants. Unfortunately, HtrA did not bind to the immobilized substrate peptide, most probably due to its short length and the possible steric hindrance. Therefore, we used the immobilized model protein substrate, β-casein, to measure binding parameters. It has to be remembered that HtrA cleaves β-casein at numerous positions; thus, it binds to this substrate at various sites with different affinity. Therefore, the presented sensorgrams (Fig. 2) show an averaged affinity of the HtrA versions towards β-casein and do not allow for the precise calculation of the binding parameters. Nevertheless, we found that ΔCysHtrA bound β-casein with a markedly higher association strength and rate than the control HtrA (Fig. 2).

### Reduction of the S-S bond does not change the secondary structure, but does affect the tertiary structure of HtrA

**Fig 3:**
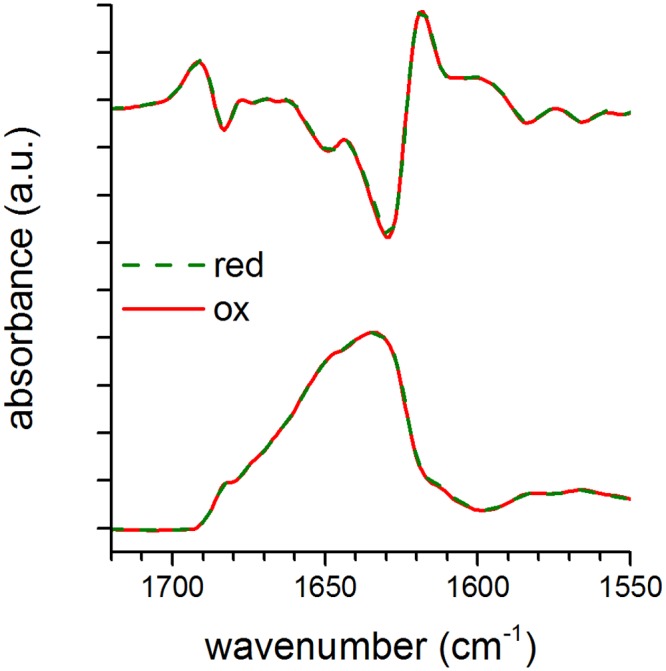
Second derivative and deconvoluted infrared absorbance spectra of oxidized and reduced HtrA-S210A at 20°C. Superimposition of HtrA-S210A reduced in the presence of DTT (red; green color) and oxidized HtrA-S210A version (ox; red color). Top: the second derivative spectra calculated over a 5-data point range (5 cm^-1^). Bottom: the deconvoluted spectra calculated with a half-band width at 19 cm^-1^ and a resolution enhancement factor of 3. At least 16 spectra were collected; a.u., arbitrary units.

To compare the secondary structures of the reduced and oxidized forms of HtrA, we undertook an FT-IR spectroscopy analysis. Since the HtrA molecule is composed mainly of the β structures, this technique provides more details than far-UV CD spectroscopy and has been successfully used to analyze the structure of HtrA [15] [24] [41]. Fig. 3 shows the superimposition of second derivative (upper panel) and deconvoluted (lower panel) spectra showing the amide I’ bands of the reduced and oxidized forms of HtrA. The assignment of band components to particular secondary structure components has been provided in our previous studies [15] [24] [41]. The spectra of HtrA in the presence of DTT (green color) overlap those of oxidized HtrA (red color); thus, the redox status of Cys57 and Cys69 does not affect the secondary structure of the protein at 20°C.

**Fig 4:**
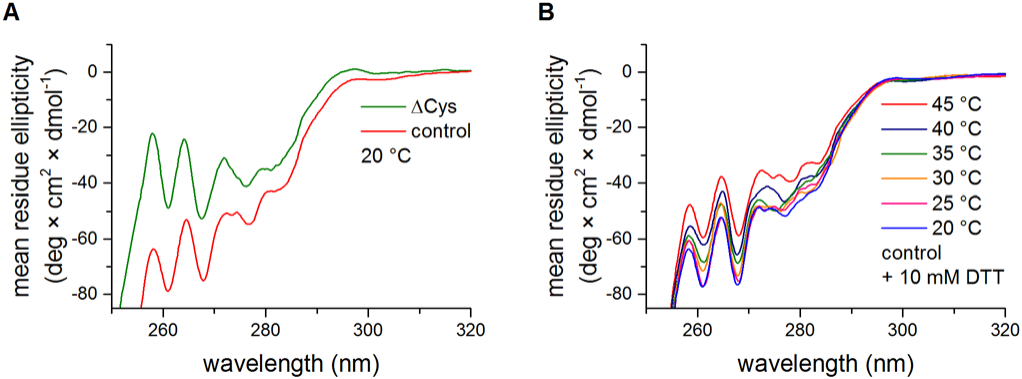
Circular dichroism analysis of HtrA. (A) Near-UV spectra of ΔCys (C57A/C69A/S210A) and oxidized control (S210A) HtrA variants at 20°C. (B) Near-UV spectra of control HtrA variant incubated in the presence of 10 mM DTT over a temperature range of 20–45°C. In each condition at least three scans were performed.

**Fig 5:**
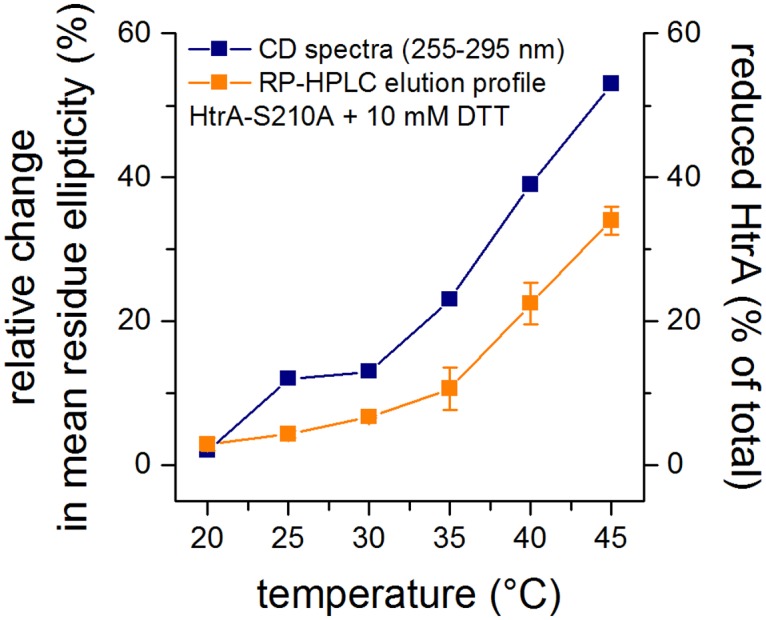
Analysis of the redox state and structural changes in the presence of DTT over a temperature range of 20–45°C. Left axis: relative change in near-UV circular dichroism (CD) ellipticity integrated at the range of 255–295 nm of HtrA-S210A in the presence of DTT (10 mM) as a fraction of ΔCys variant (HtrA-C57A/C69A/S210A) ellipticity subtracted from HtrA-S210A ellipticity in the absence of DTT. Right axis: amount of HtrA-S210A reduced by DTT as a fraction of total HtrA-S210A in the sample, as calculated from integrated area under reversed-phase high performance liquid chromatography (RP-HPLC) peaks. The error bars represent the standard deviation values from at least two independent measurements.

Infrared spectroscopy does not provide any direct information about changes of the tertiary or quaternary structure of a protein. Therefore, we performed a near-UV CD spectroscopy analysis of the ΔCys and the control HtrA. In the range of 250–330 nm, the CD signals originate mainly from aromatic residues and cysteine. The HtrA molecule is rich in aromatic amino acids (14 Phe and 5 Tyr) and its near-UV CD spectrum, especially within the range 255–270 corresponding to Phe absorption bands, was shown to be indicative of tertiary structure changes [24]. The actual shape and magnitude of the near-UV CD spectrum of a protein depends on the number of each type of aromatic amino acid present, their mobility, the nature of their environment (H-bonding, polar groups and polarizability) and their spatial disposition in the protein [39]. Disulfide bonds give rise to weak broad negative signals near 250 nm [42]. The near-UV spectra of both HtrA variants were very similar in their shapes, indicating that the tertiary structure elements were preserved (Fig. 4A and S1 Fig.). However, the intensity of the signal in the case of the oxidized protein was larger. A small part of the ΔCys HtrA signal reduction could be due to the loss of the spectral contribution from the cysteine, but it may also suggest that the protein containing its S-S bond has a more compact structure [43]. To ensure that the changes of the intensity of the CD signal were not due to erroneously estimated protein concentrations, we collected the near-UV CD spectra of control HtrA treated with DTT (Fig. 4 B). At low temperatures (20–25°C), the spectra were very similar to those recorded in the absence of DTT. As temperatures rose, the intensities of signals decreased, indicating the occurrence of S-S bond disruption. As the CD spectra of both HtrA variants were not markedly changed upon temperature rise (20→45°C), the observed shift should be attributed to the reduction of the disulfide within the HtrA molecule. To prove this, the HtrA samples, which were treated with DTT under conditions mimicking those used for the CD measurements, were separated via RP-HPLC. Reduced and oxidized HtrA versions (HtrA_red_ and HtrA_ox_, respectively) were eluted as two separate peaks, at approximately 5.2 ml and 5.3 ml, respectively (S2 Fig.). As can be seen in Fig. 5, at low temperatures (20–25°C) HtrA was very poorly reduced by DTT. With the rise in temperature, the fraction of the reduced HtrA grew and the changes occurred in parallel to differences in the mean residue ellipticity values that occurred due to the reduction of HtrA (Fig. 4 B).

However, the extent of the difference between the intensities of CD signals in the presence or in the absence of DTT were not equal to the extent of the reduction of HtrA (Fig. 5). Thus, the upward shift of the CD spectrum of ΔCys HtrA in respect to that of oxidized control HtrA (Fig. 4 and S1 Fig.) most probably results from the combination of two effects: (1) a reduction of the disulfide bond and (2) subtle overall structural changes induced by disruption of the S-S bond.

### Thermal denaturation of the reduced and oxidized forms of HtrA

**Fig 6:**
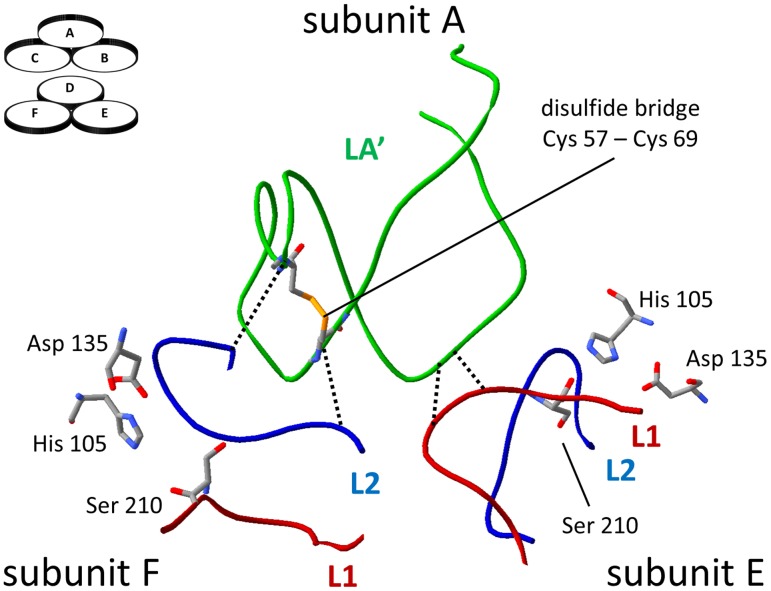
Interactions between LA’, L1, and L2 loops. The LA loop model in inactive conformation (PDB entry: 1ky9) according to [23]. The positions of subunits A, E, and F within the hexamer are shown in the left top corner. His105, Asp135, and Ser210 constitute the catalytic triad. The selected contacts between the loops are shown as dotted lines. The picture was generated with the aid of Swiss-PdbViewer 4.1 [44].

**Fig 7:**
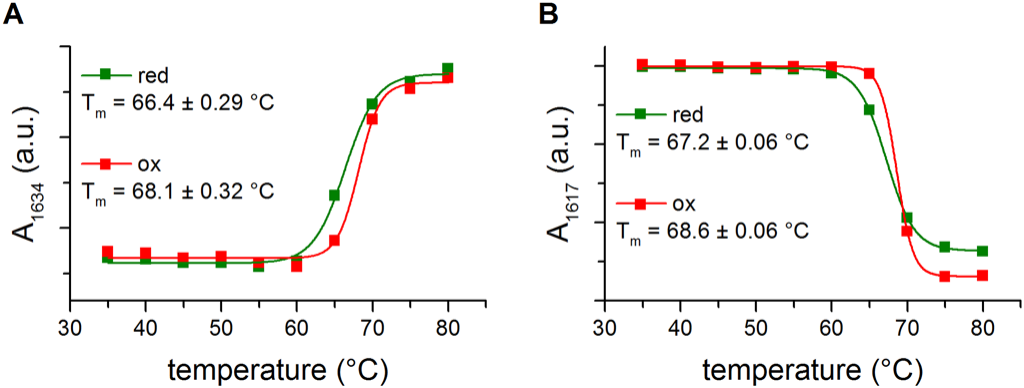
Thermal denaturation curves of reduced and oxidized HtrA-S210A. (A) 1634 cm^-1^ peaks—loss of β structures. (B) 1617 cm^-1^ peaks—gain of random coil structures; red—HtrA reduced in the presence of DTT, ox—oxidized HtrA. Curves were obtained by monitoring the second derivative of infrared spectra. T_m_ (melting temperature) values were calculated as described in “Materials and Methods”. In each temperature at least 16 spectra were collected; a.u., arbitrary units.

Disulfide bonds are known to increase the conformational stability of many proteins. However, in the case of HtrA the Cys57-Cys69 disulfide connects parts of a mobile loop LA (Fig. 6) and does not link any of the secondary structures. To assess the influence of the redox state of Cys57-Cys69 on HtrA thermal stability, we subjected the reduced and oxidized versions of HtrA to thermal denaturation. When a protein is heated above its characteristic thermal stability point (the melting temperature, T_m_), it undergoes unfolding and aggregation and both events may serve as determinants of protein stability. The T_m_ values based on unfolding (the decrease of a signal at 1634 cm^-1^ corresponding to β-sheets) or aggregation (the appearance of a signal at 1617 cm^-1^) were similar for both variants (in the range of 66–68°C) (Fig. 7 A and B).

**Fig 8:**
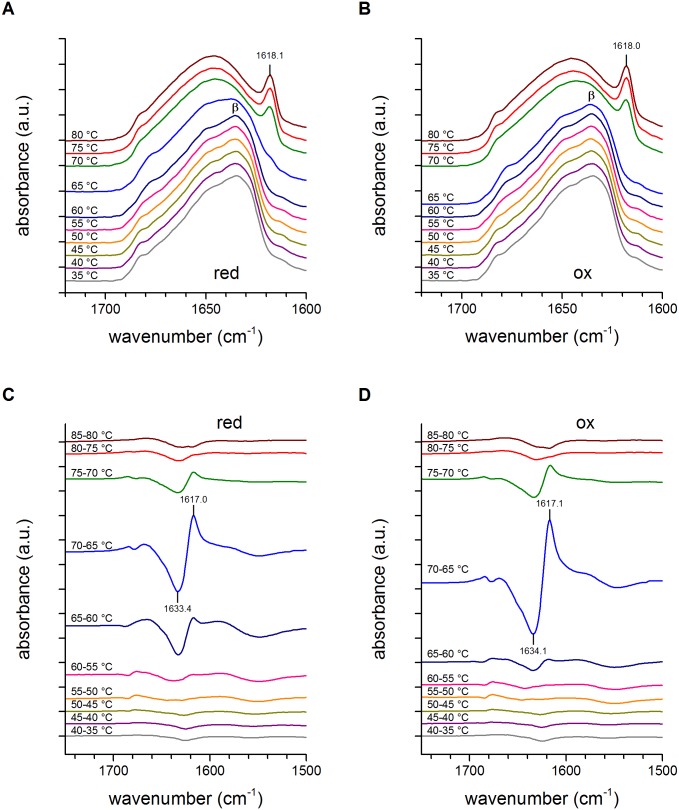
Deconvoluted and difference spectra of reduced and oxidized HtrA as a function of temperature. (A, B) deconvolued spectra of red (reduced in the presence of DTT) and ox (oxidized) HtrA-S210A versions collected in 5°C steps. (C, D) difference spectra of red and ox HtrA-S210A versions between two original absorbance spectra collected at different temperatures (5°C steps). The bands at about 1634 and 1617 cm^-1^ correspond to β-sheet and random coil structures, respectively. In each temperature at least 16 spectra were collected; a.u., arbitrary units.

The calculated T_m_ values were in agreement with the temperature-dependent profile of the deconvoluted (Fig. 8 A and B) and difference spectra (Fig. 8 C and D) of the reduced and oxidized forms of HtrA. The maximum of denaturation can indeed be observed between 65 and 70°C for both proteins. However, in the case of the reduced protein a marked change of the shape of the spectrum between 60 and 65°C is visible, indicating that HtrA_red_ also lost a significant part of the secondary structure in this temperature range. More detailed information on thermal denaturation can be obtained by difference spectra (Fig. 8 C and D) [15] [41] [45]. The negative 1634.1 cm^-1^ broad band indicates a lower content of secondary structural elements in the sample at 70°C with respect to the sample at 65°C and, in turn, indicates the unfolding (denaturation) of the protein induced by the increased temperature. The positive 1617.1 cm^-1^ band was due to protein aggregation brought about by protein denaturation [15] [41] [45]. In the case of HtrA_red_, the denaturation and aggregation events covered a broader range of temperatures, since at 65–60°C the spectrum of HtrA_red_ showed larger positive and negative bands than that of HtrA_ox_. In particular, the onset of denaturation can be observed at 65–60°C and at 60–55°C for HtrA_ox_ and HtrA_red_, respectively, as indicated by the significant negative bands in the amide I’ region (1700–1600 cm^-1^). Thus, although the midpoints of denaturation were similar for both HtrA variants, unfolding of HtrA_red_ started at a lower temperature in respect to HtrA_ox_. Since the secondary structures of the two protein samples were the same, it is possible that the different thermal denaturation behavior is due to the different tertiary and/or quaternary structure assumed by the reduced HtrA.

### Fluorescence monitored structural changes within the LA loop due to the lack of its disulfide bridge

We obtained two proteolytically inactive single-tryptophan HtrA versions, HtrA-C57A/F63W/C69A/S210A (ΔCysW variant) and HtrAF63WS210A (oxidized control variant). The presence of Trp at this position should not disturb the LA structure, as the Trp side chain can form hydrophobic interactions with the remaining Phe residues within the hydrophobic cluster formed by the LA loop [23]. We have also shown that the F63W substitution does not disturb either HtrA’s secondary structure, or its thermal stability [24]. Thus, we expected Trp63 to serve as a good indicator of putative structural changes in this region of the LA loop. We analyzed the fluorescence properties of the Trp indole rings in both variants. First of all, we monitored the positions of fluorescence emission maxima (λ_max_) which are indicative of the polarity of the microenvironment of Trps [46]. We found that the λ_max_ values of the corrected fluorescence emission spectra differed significantly in both HtrA variants. The mutations C57A/C69A caused a significant (9 nm) red shift of λ_max_ in respect of the control protein, as measured at 20°C (351.7 ± 0.5 and 342.7 ± 0.5 nm, respectively), which indicates that Trp63 in the ΔCys mutant is in a definitely more polar environment. The observed differences in the spectral properties of the Trps suggest that the mutation ΔCys caused a significant alteration of the LA loop structure.

**Fig 9:**
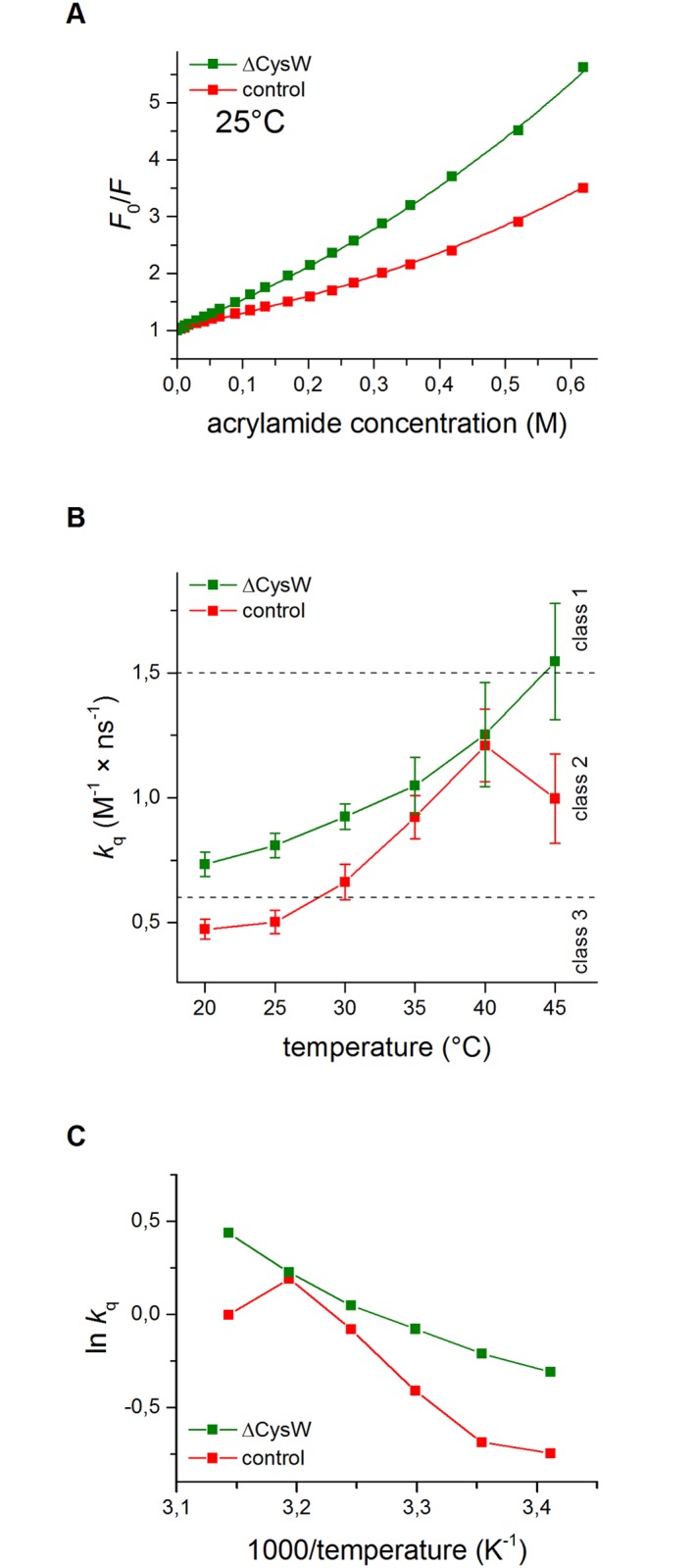
Quenching of the tryptophan fluorescence of the single-Trp HtrA variants by acrylamide. (A) typical Stern-Volmer quenching plots of ΔCysW (C57A/C69A/F63W/S210A) and oxidized control (F63W/S210A) HtrA variants at 25°C. *F*_0_ and *F* are the fluorescence intensities in the absence and presence of acrylamide, respectively. (B) the bimolecular quenching constant (*k*_q_) values for HtrA variants as a function of temperature. The exposition classes are given according to [49] and [50]. (C) Arrhenius plots for the *k*_q_ values from panel B. All data were obtained as described in [24] and correspond to mean ± standard deviation values of at least three different experiments.

To gain more information about the structural changes caused by the lack of an S-S bond, we performed steady-state fluorescence quenching experiments at temperatures of 20–45°C. This technique provides information on the accessibility and degree of exposure of the Trp side chains within a protein [47]. As a quencher we used acrylamide, a molecule which is able to penetrate into a protein and quenches both the exposed and buried tryptophan residues, primarily via a collisional mechanism [47]. The quenching data was plotted according to the Stern-Volmer equation [48]. Typical Stern-Volmer quenching plots of the single-Trp HtrA mutants at 25°C are shown in Fig. 9 A. The quenching plots were nearly linear at low acrylamide concentrations. At high acrylamide concentrations, a small upward curvature was observed suggesting the occurrence of static quenching. It seems that the apparent static component is due to the quencher being adjacent to the fluorophore at the moment of excitation (“sphere of action”). The close proximity of a quencher and a fluorophore may cause immediate quenching of fluorescence upon excitation. Therefore, the plots were analyzed in terms of both dynamic (*K*_sv_) and sphere-of-action quenching (*V*) (Table 3).

**Table 3:** Comparison of the fluorescence properties of the single-Trp HtrA variants.

	ΔCysW (HtrA-C57A/C69A/F63W/S210A)				oxidized control (HtrA-F63W/S210A)			
temperature	*K*_SV_ [M^-1^]	*V* [M^-1^]	*k*_q_ [M^-1^ × ns^-1^]	class	*K*_SV_ [M^-1^]	*V* [M^-1^]	*k*_q_ [M^-1^ × ns^-1^]	class
20°C	3.29 ± 0.22	0.82 ± 0.06	0.73 ± 0.05	2	1.57 ± 0.13	0.20 ± 0.07	0.47 ± 0.04	3
25°C	3.41 ± 0.21	1.18 ± 0.06	0.81 ± 0.05	2	1.54 ± 0.14	1.09 ± 0.07	0.50 ± 0.05	3
30°C	3.61 ± 0.20	1.34 ± 0.05	0.92 ± 0.05	2	1.86 ± 0.20	0.69 ± 0.08	0.66 ± 0.07	2
35°C	3.77 ± 0.41	1.74 ± 0.12	1.05 ± 0.11	2	2.41 ± 0.23	0.76 ± 0.08	0.92 ± 0.09	2
40°C	4.12 ± 0.68	2.53 ± 0.24	1.25 ± 0.21	2	2.91 ± 0.35	0.77 ± 0.15	1.21 ± 0.15	2
45°C	4.55 ± 0.69	2.05 ± 0.24	1.54 ± 0.23	1	2.26 ± 0.41	1.55 ± 0.17	1.00 ± 0.18	2

*K*_SV_—Stern-Volmer (dynamic quenching) constant, *V*—static (sphere of action) quenching constant, *k*_q_—bimolecular quenching constant. The Trp residues according to [49] and [50] are classified as: (1) = exposed [1.5 < *k*_q_ < 5], (2) = moderately exposed to the solvent [0.6 < *k*_q_ < 1.5], (3) = moderately buried [0.2 < *k*_q_ < 0.6].

To directly compare the accessibility of the fluorophores to the quencher, the bimolecular quenching constants, *k*_q_, were calculated (*k*_q_ = *K*_sv_/τ_0_). The mean fluorescence lifetimes, τ_0_ (S1 Table), were only used for the calculation of the *k*_q_ values. The results are shown in Table 3 and in Fig. 9 B. As can be seen in Fig. 9 B, at low temperatures the Trp residue of HtrA with the S-S bond was moderately buried (as shown previously in [24]), whereas the Trp of the ΔCysW variant was moderately exposed (according to [49] and [50]), even at temperatures as low as 20°C and the *k*_q_ values differed significantly. Generally, Trp in ΔCysW HtrA (representing a reduced form) was more exposed to the solvent in respect to the oxidized form at temperatures up to 40°C. At this temperature, Trp residues of both HtrA forms were equally accessible to the quencher. Interestingly, at 45°C, the *k*_q_ value rapidly decreased in the case of the control HtrA (oxidized), whereas the *k*_q_ value of the ΔCysW variant kept rising.

The Arrhenius plot (Fig. 9 C) enables us to draw further conclusions concerning the *k*_q_ values. A discontinuity in the plot may reflect a conformational change in the protein. The plot of the control protein exhibits two prominent discontinuities of the Arrhenius plot: a slightly upwards one at 25°C and a strong downwards one at 40°C. Presumably, at these transition temperatures conformational changes occur in the LA loop in the microenvironment of the Trp63 residue. One leading to a greater exposition to a solvent (at 25°C), the other (at 40°C) associated with a restriction of access to the Trp63 residue (Fig. 9 B). Interestingly, in the case of cysteineless HtrA no significant discontinuities were observed. Most probably, the LA loop lacking the S-S bridge adopts a structure which behaves differently upon temperature shift.

### The Cys57-Cys69 S-S bond affects the oligomerization of HtrA

**Fig 10:**
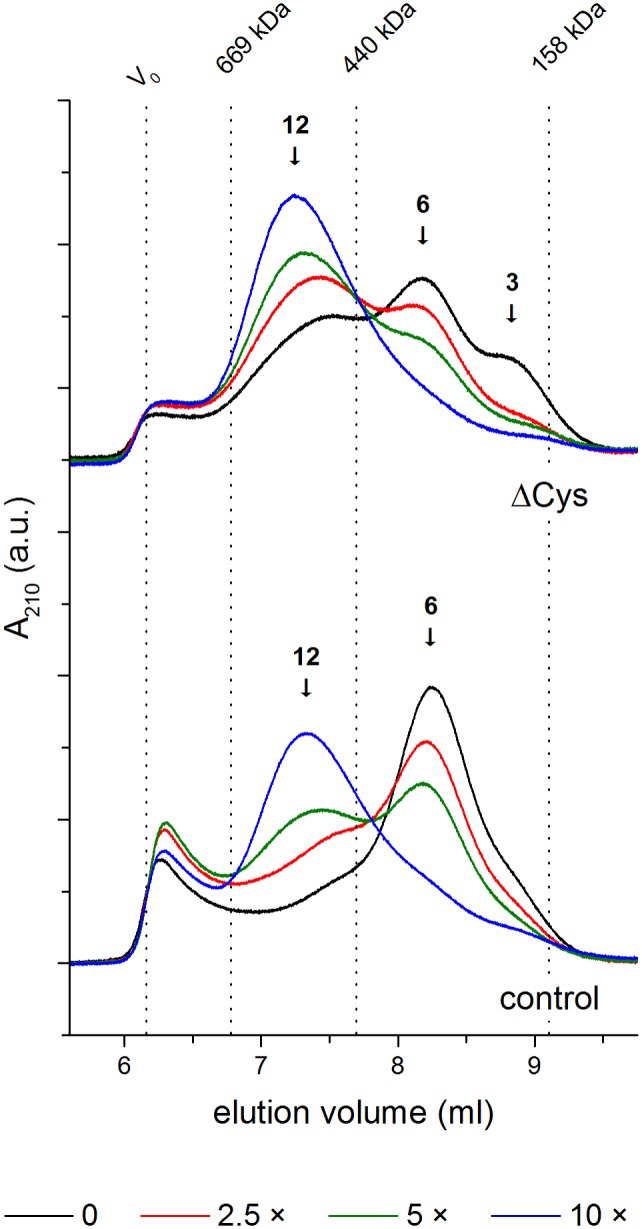
The oligomeric states of the HtrA protein variants in the presence or absence of a peptide substrate. ΔCys (C57A/C69A/S210A) and oxidized control (S210A) HtrA variants were incubated with a 2.5-, 5-, or 10-fold molar excess of the 22-peptide NWVSAAKFESTDGSTDYGIYQV (2.5 ×, 5 ×, or 10 ×, respectively) or without ligand (0), subjected to cross-linking with bis[sulfosuccinimidyl] suberate (BS^3^), and then analyzed using size exclusion chromatography as described in “Materials and Methods”. The void volume (V_0_) and elution volumes of molecular weight standards (Bio-Rad) used for column calibration are shown as vertical dotted lines. The positions of HtrA dodecamers (12), hexamers (6), and trimers (3) are indicated with arrows. A representative elution profile is shown; a.u., arbitrary units.

To check if the structural changes driven by the ΔCys mutation affect the oligomerization status of HtrA, we monitored the ability of both HtrA variants to assemble into various oligomeric forms applied to SEC. In accordance with previously published data, control HtrA was mainly eluted at the position corresponding to the hexameric form with a small fraction of large oligomeric forms (possibly 24-mers) [40] [23]. The elution profile of ΔCys HtrA was markedly different. The protein migrated in the resin as a mixture of trimers, hexamers (dominant), 12-mers and a small amount of large oligomers (Fig. 10, upper panel). This finding indicates that the lack of an S-S bond decreases the stability of HtrA inter-subunit connections.

The addition of a 22-peptide ligand leads to the reassembly of the HtrA oligomers. In particular, hexamers dissociate to trimers which subsequently form high-order oligomeric forms [21]. As can be seen in Fig. 10, both HtrA variants assembled into 12-mers in the presence of a 22-peptide; however, they showed markedly different dependence on substrate concentration. At low concentrations of the peptide (2.5-fold molar excess), the ratio of 12/6-mers was much higher in the case of ΔCys HtrA than in the case of the oxidized control HtrA. Moreover, we observed that the peaks corresponding to trimers tend to disappear following the addition of the peptide. Thus, the lack of the S-S bond does not disturb the formation of high-order oligomeric forms; just the opposite—it seems to facilitate dissociation of the resting state hexamers and further assembly to 12-mers.

### HtrA can be converted into a reduced state by human Trx1

**Fig 11:**
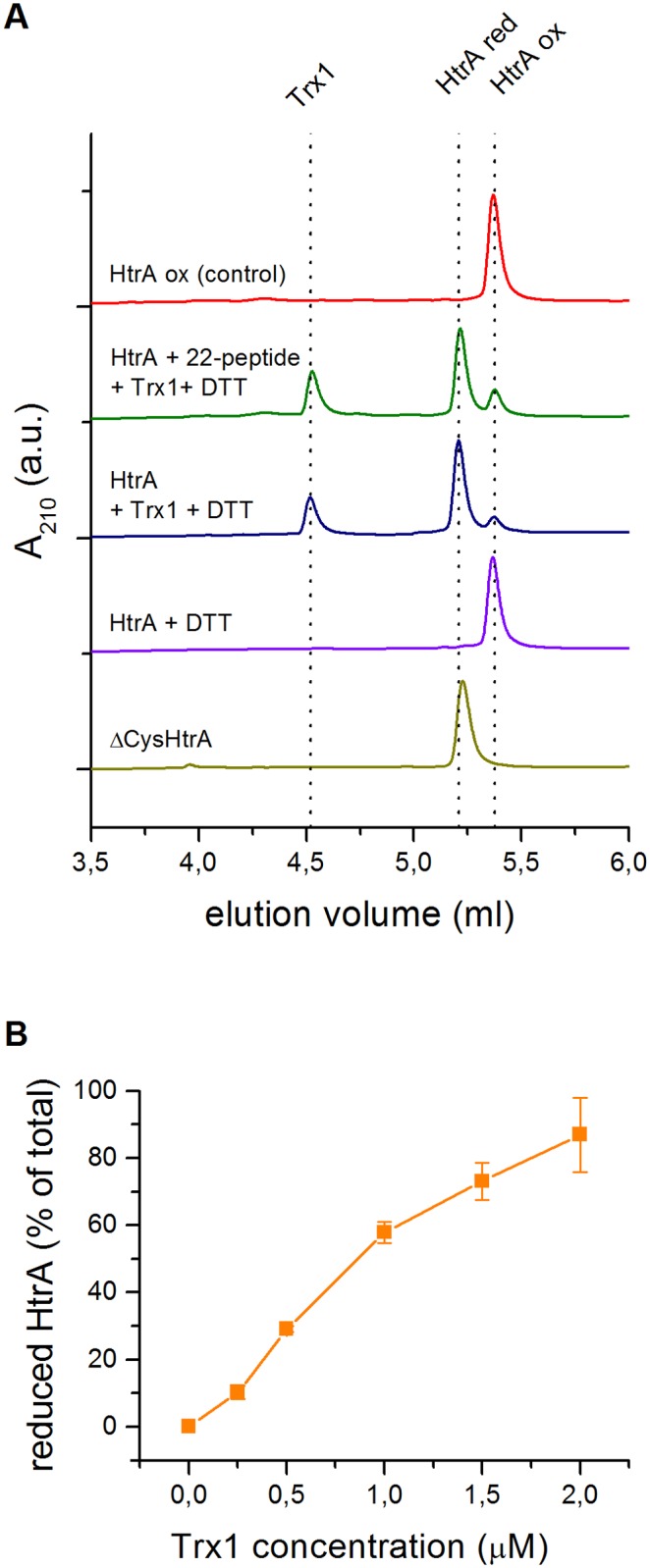
Enzymatic reduction of HtrA by human Trx1. (A) Reversed-phase high performance liquid chromatography (RP-HPLC) analysis of HtrA-S210A redox state depending on the presence of Trx1, DTT, or 22-peptide (10-fold molar excess) in comparison to ΔCys (HtrA-C57A/C69A/S210A) variant. The elution volumes of Trx1, HtrA red (the reduced form or cysteine-less variant), and HtrA ox (the oxidized form) are shown as vertical dotted lines. A representative elution profile is shown; a.u., arbitrary units. (B) fraction of HtrA-S210A reduced by Trx1 over 15 minutes at 37°C as a fraction of total HtrA-S210A in the sample, as calculated from the integrated area under RP-HPLC peaks. The error bars represent the standard deviation values from at least two independent measurements.

There is a growing evidence that the thioredoxin (Trx) system can modulate the activity of various extracellular proteins via a reduction of their disulfide bonds [51]. Since a fraction of HtrA has been shown to be secreted outside the bacterial cell [29], we checked if HtrA can be a substrate for human Trx1. Therefore, we incubated HtrA_ox_ with different concentrations of Trx1 in the reducing environment and analyzed samples by RP-HPLC to evaluate the contents of oxidized and reduced HtrA fractions (Fig. 11). ΔCys HtrA was eluted at a very similar position to that of the HtrA_red_; thus, it was used as a reference for the reduced form. As can be seen in Fig. 11 B and S3 Fig., HtrA was reduced by Trx1 in a concentration dependent fashion: an increase in the content of Trx1 resulted in a more efficient reduction of HtrA. The presence of a substrate peptide did not affect the catalysis of the S-S bond reduction by Trx1. As has already been mentioned in this paper, HtrA is not easily reduced by DTT *in vitro*. Therefore, in the absence of Trx1 the fraction of reduced HtrA was not observed, despite the presence of 2 mM DTT (Fig. 11 A).

## Discussion

Regulation of the HtrA protease is a very complicated process, which is still not fully understood. Conversion of the inactive, resting state into an active protease requires large conformational changes and this can be accomplished via allosteric and/or temperature dependent modes. Structural rearrangements lead to the uncovering and proper organization of the active center, and are accompanied by reassembly of the hexamer to higher-order oligomers [25].

We have previously shown that the proteolytic activity of HtrA can be stimulated at low temperatures (20–25°C) by a removal of the Cys57-Cys69 S-S bond (either by reduction or mutation) [17]. The location of this disulfide in the regulatory loop LA suggests that its reduction might have an impact on HtrA activity via modulation of LA structure, mobility and/or interactions formed by the loop’s residues. In our recent study, we demonstrated that mutations in the LA loop disturbing the intersubunit hydrophobic or hydrophilic connections within the hexamer caused stimulation of the proteolytic activity of HtrA [23]. Reduction of the S-S bond may lead to destabilization of both types of interactions, as the mobility of the LA loop should increase. This assumption was confirmed by the observation that a Trp residue introduced into the LA loop at position 63 was more exposed to the solvent in ΔCys HtrA in respect to HtrA_ox_. Consequently, the hydrophobic cluster comprising residue 63 must have been affected by the lack of the disulfide connection. Also, the position of Gln70 in respect to Asn206, Arg207 and Asn209 (L1 loop of the opposite trimer) should be no more stabilized in HtrA_red_. Thus, the reduction of the disulfide within LA may facilitate the liberation of the active center elements from the inhibitory influence of this loop. The reduction of the LA loop S-S bond had more extensive consequences. Although the spectroscopic analysis of HtrA did not reveal significant changes in the protein structure, the thermal denaturation behavior of the HtrA_red_ molecule was different with the onset of unfolding at lower temperatures. Moreover, the ΔCys HtrA oligomers were less stable in the absence of a substrate in respect to HtrA_ox_. Consequently, a markedly lower concentration of a substrate peptide was sufficient to shift the oligomer equilibrium towards a dodecameric form.

All these structural features had a significant impact on the kinetics of substrate turnover. HtrA is an allosteric enzyme showing a homotropic positive cooperativity upon the binding of a substrate molecule [37] [40]. Although both HtrA versions, ΔCys and oxidized, were characterized by the similar Hill constant and apparent *K*’ values, they differed in terms of *V*_max_, with a markedly higher value calculated for ΔCys HtrA. Normally, *K*’ represents an apparent substrate affinity of an enzyme to a tested substrate. However, in the case of HtrA the situation is more complicated. As already discussed in the recent study by Kim and Sauer [37], the *K*’ value is the resultant of the strength of the substrate-oligomer binding, the energetic costs of the transition of the enzyme from the inactive to the active conformation, and the costs of the oligomer reassembly. Also, the affinity of various HtrA oligomeric forms towards a peptide substrate does not seem to be equal. It has been suggested that the dodecameric form of HtrA binds a substrate more tightly than do the lower-order oligomers [37]. We have demonstrated in this study that the preparation of ΔCys HtrA formed higher-order oligomeric forms more easily and contained a substantial amount of dodecamers even in the absence of a substrate. Therefore, we expected that the effective affinity of ΔCys HtrA to its substrate should be higher than that of HtrA_ox_. Indeed, the SPR analysis of the HtrA-substrate interactions confirmed this assumption and indicated that the apparent affinity of HtrA to a model protein substrate (β-casein) was significantly higher in a version lacking its S-S bond. As a consequence, reduction of the S-S bond facilitates the transition of an HtrA molecule into a form that binds and cleaves its substrates more efficiently.

Summing up, a decreased thermal stability and a lower stability of the hexamer in the case of HtrA_red_ confirms the importance of the LA loop in the stabilization of HtrA molecules. The reduction of the S-S bond most probably results in a greater flexibility of the part protruding into the opposite trimer. It is likely that this feature facilitates the remodeling of HtrA molecules into an active form.

The reduced form of HtrA appears in the periplasm when the cellular redox equilibrium becomes disturbed, either due to growth in the reducing environment, or as a consequence of the incorrect functioning of the Dsb system [19] [18]. In any case, the formation of S-S bonds in the periplasmic proteins is disturbed and there is an increased demand for the HtrA function to prevent accumulation of incorrectly folded proteins in the bacterial envelope.

*E*. *coli* is a common inhabitant of the gastrointesitinal tract of humans and animals. The redox potential of intestines may reach values ranging from-150 mV (small intestine) to as low as-300 mV in the colon. These values are variable and depend on the actual diet and the metabolism of the gut microbiota [52] [53]. Thus, *E*. *coli* cells residing in the intestines are exposed to both oxidizing and reducing conditions, and in the latter case the periplasm may encounter a reducing stress.

Recently, it has been demonstrated that certain gastrointestinal Gram-negative pathogens, including enteropathogenic *E*. *coli*, secrete a portion of HtrA extracellularly [29]. In this case, the redox state of HtrA should be dependent on the activity of the secreted oxidoreductases of a host, e.g. Trx1. Trx1 is efficiently expressed by the gastric, illeal and colonic mucosa. Trx1 has been shown to efficiently reduce human β-defensin 1 at the surface of the intestine mucosa [52] [54]. In this study, we have demonstrated that HtrA can be a substrate for Trx1. Hence, the reduction of the S-S bond may represent an additional mechanism to stimulate the proteolytic activity of the extracellular fraction of HtrA. As the proteolytic cleavage of E-cadherin by HtrA is regarded as an important step of pathogenesis, it is tempting to speculate that the reduction of HtrA increases the efficiency of the disruption of epithelial barriers by bacteria.

**Fig 12:**
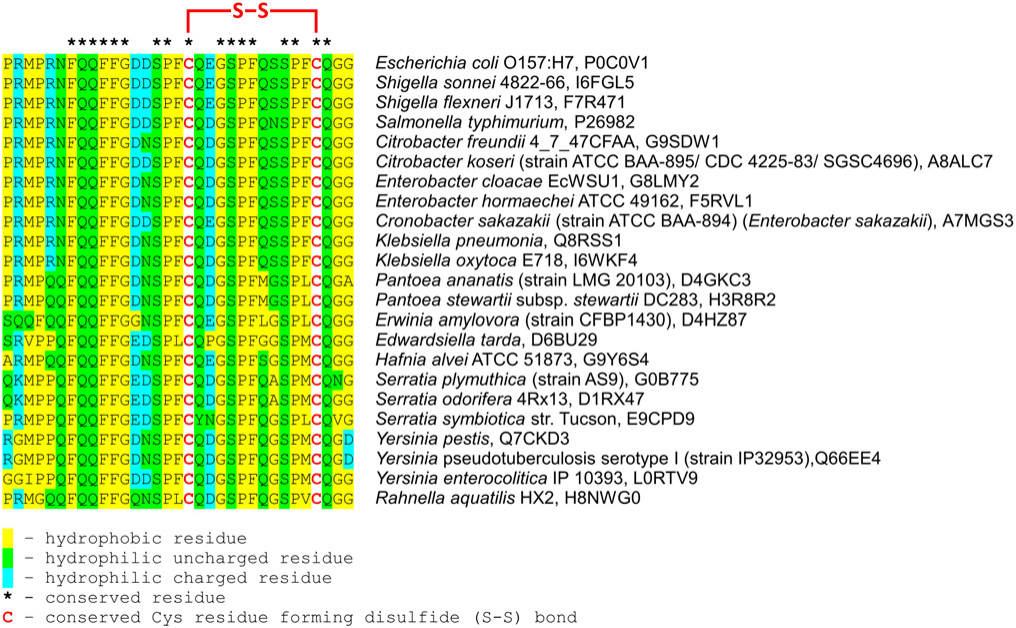
Comparison of evolutionarily conserved sequence within the LA loop surrounding disulfide bonds in the Enterobacteriaceae family. The sequence alignment to residues 40–72 from *E*. *coli* HtrA generated by Clustal 2.1 [56]. The classification of residue hydrophobicity is according to [57].

The structure of the LA loop, including the position of the disulfide bond, seems to be typical across the Enterobacteriaceae family (Fig. 12). The positions of the hydrophobic and hydrophilic as well as cysteine residues are strongly conserved. On the other hand, the presented above characteristics of the LA loop applies only to the HtrA (DegP) proteins from Enterobacteriaceae. The LA loops of HtrAs originating from the other Gram-negative bacterial families show very low homology with LA from Enterobacteriaceae. They are in general shorter and do not contain a disulfide bond. Thus, the proposed regulatory mechanism is limited to HtrAs from Enterobacteriaceae. However, the Enterobacteriaceae family of bacteria comprises both pathogens or commensals of the digestive system (e.g. *E*. *coli*, *S*. *flexneri*, *Salmonella enterica*, *Enterobacter cloacae*) and bacteria that reside outside the gastrointestinal tract (e.g. *Klebsiella pneumoniae*, *Yersinia pestis*, *Yersinia pseudotuberculosis*, *Enterobacter hormaechei*, *Cronobacter sakazakii*), as well as plant pathogens (e.g. *Erwinia amylovora*). Several species are opportunistic pathogens and can be found in water or soil, but can infect humans as well (e.g. *Citrobacter freundii*, *Klebsiella oxytoca*). Summing up Enterobacteriaceae can be found in a wide variety of habitats [55]. In all cases bacteria may encounter stressful conditions, including redox imbalance. Thus, the redox-dependent regulation of the proteolytic activity of HtrA may be important for better survival of stress and adaptation to changing environmental conditions. The contribution of HtrA to the virulence of many bacterial species has been proven and HtrA has been designated a potentially attractive therapeutic target. Therefore, an understanding of the precise mechanism of HtrA functioning and its regulation is of special importance. We believe that the results of this study provide a solid contribution to this field and allow for a better understanding of the regulatory role of the LA loop in HtrA from *E*. *coli*, a model bacterium of the Enterobacteriaceae family.

## Supporting Information

S1 Fig Circular dichroism analysis of HtrA. Near-UV spectra of HtrA variants across a temperature range of 20–45°C. (A) ΔCys (HtrA-C57A/C69A/S210A) variant. (B) oxidized control (HtrA-S210A) variant. In each condition at least three scans were performed.
(TIF)

S2 Fig Analysis of HtrA redox state. Control (S210A) and ΔCys (C57A/C69A/S210A) or control HtrA variants in the presence of 10 mM DTT were incubated at a given temperature (within the range of 20–45°C) and applied on an RP-HPLC column as described in “Materials and Methods”. The elution volumes of HtrA red (the reduced form or cysteine-less variant) and HtrA ox (the oxidized form) are shown as vertical dotted lines. A representative elution profile is shown; a.u., arbitrary units.
(TIF)

S3 Fig Analysis of HtrA redox state in the presence of Trx1. 20 μM of control (S210A) and ΔCys (C57A/C69A/S210A) or control HtrA variants in the presence of 0.25–1.50 μM Trx1 were incubated at 37°C for 15 minutes and applied on an RP-HPLC column as described in “Materials and Methods”. The elution volumes of Trx1, HtrA red (the reduced form or cysteine-less variant), and HtrA ox (the oxidized form) are shown as vertical dotted lines. A representative elution profile is shown; a.u., arbitrary units.
(TIF)

S1 Table Fluorescence lifetime properties of the Trp residues introduced into HtrA. (DOC)
