# Supplementary figures and images for: Analysis of the Link between the Redox State and Enzymatic Activity of the HtrA (DegP) Protein from Escherichia coli
Source: PLoS One. 2015 Feb 24;10(2):e0117413. doi: 10.1371/journal.pone.0117413 (PMC4339722; doi:10.1371/journal.pone.0117413)

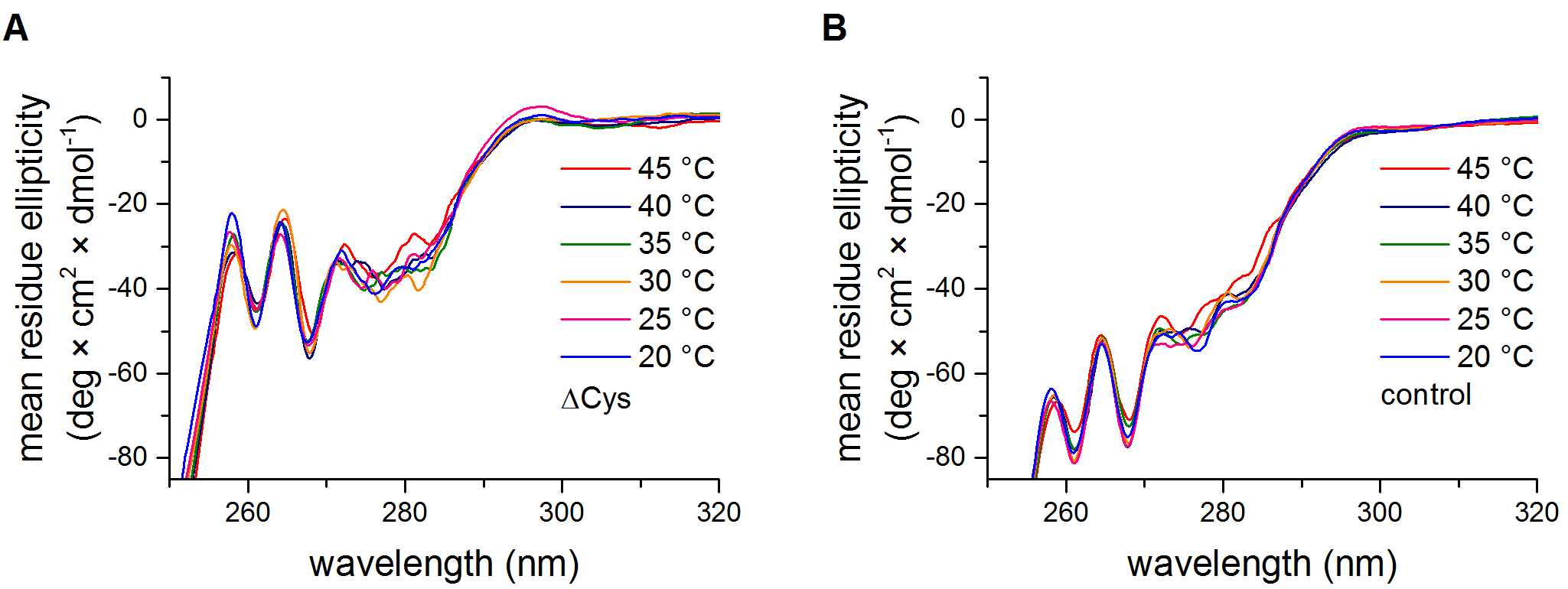

Supplement: S1 Fig — Near-UV spectra of HtrA variants across a temperature range of 20–45°C. (A) ΔCys (HtrA-C57A/C69A/S210A) variant. (B) oxidized control (HtrA-S210A) variant. In each condition at least three scans were performed. (TIF) [file pone.0117413.s001.tif]

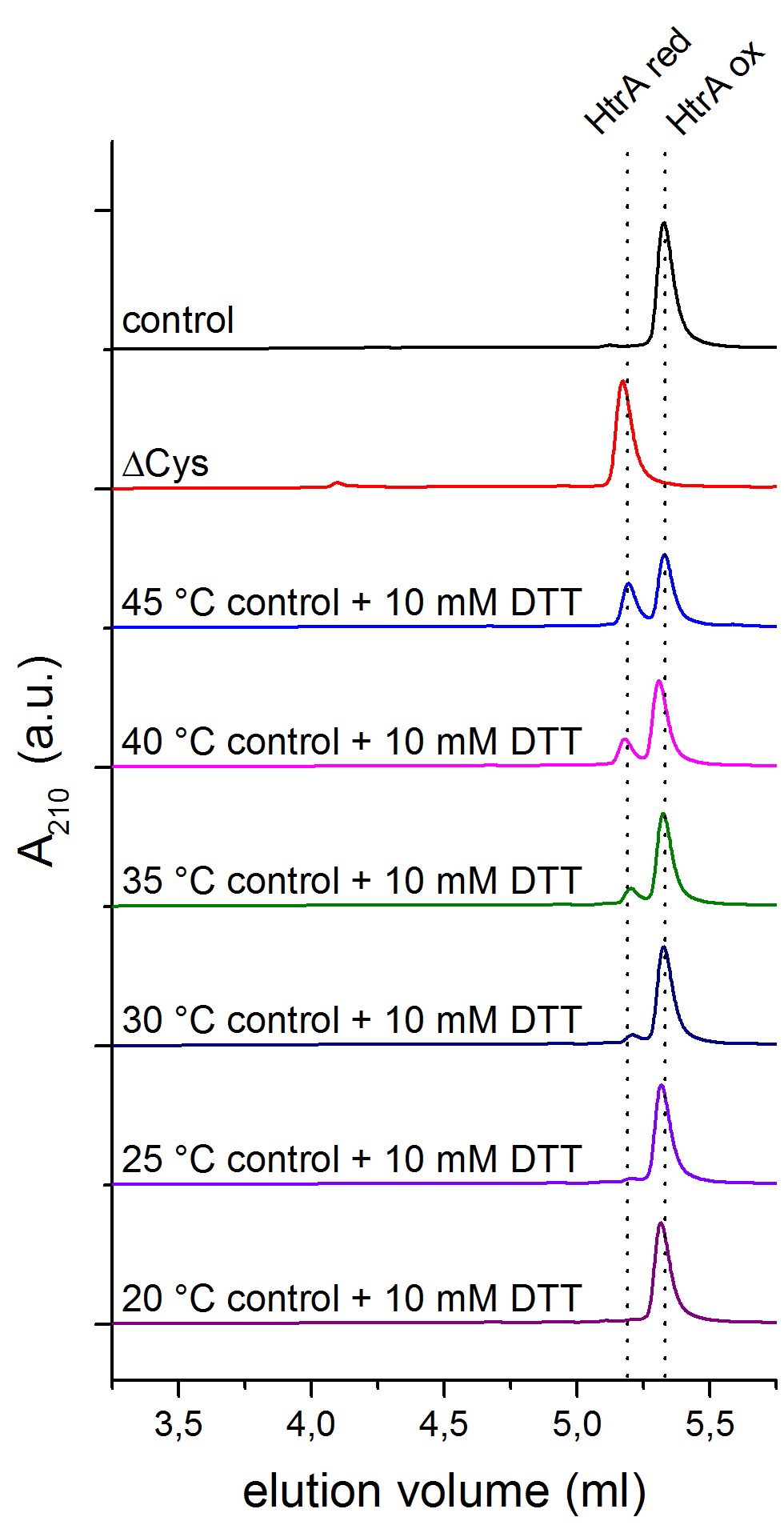

Supplement: S2 Fig — Control (S210A) and ΔCys (C57A/C69A/S210A) or control HtrA variants in the presence of 10 mM DTT were incubated at a given temperature (within the range of 20–45°C) and applied on an RP-HPLC column as described in “Materials and Methods”. The elution volumes of HtrA red (the reduced form or cysteine-less variant) and HtrA ox (the oxidized form) are shown as vertical dotted lines. A representative elution profile is shown; a.u., arbitrary units. (TIF) [file pone.0117413.s002.tif]

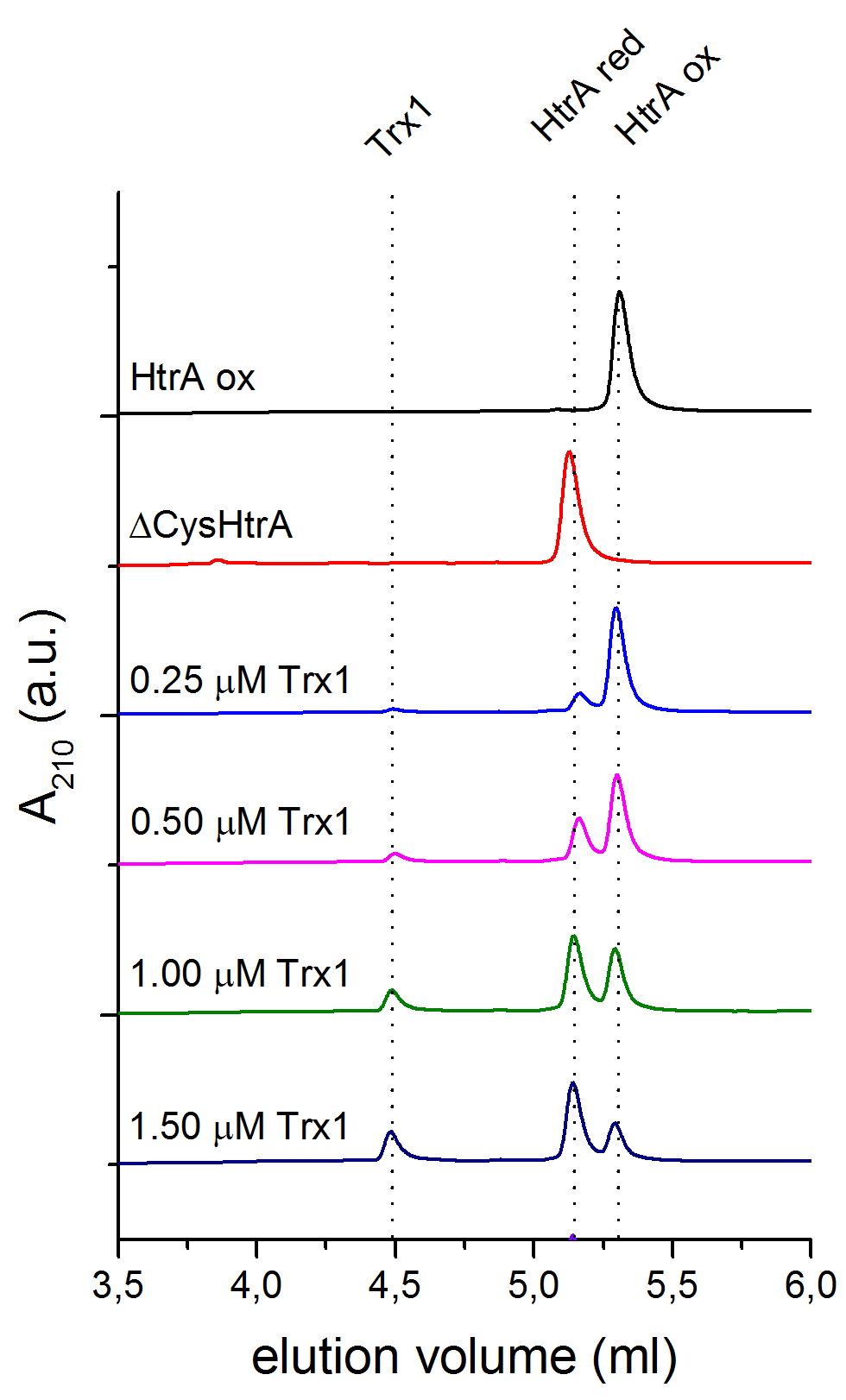

Supplement: S3 Fig — 20 μM of control (S210A) and ΔCys (C57A/C69A/S210A) or control HtrA variants in the presence of 0.25–1.50 μM Trx1 were incubated at 37°C for 15 minutes and applied on an RP-HPLC column as described in “Materials and Methods”. The elution volumes of Trx1, HtrA red (the reduced form or cysteine-less variant), and HtrA ox (the oxidized form) are shown as vertical dotted lines. A representative elution profile is shown; a.u., arbitrary units. (TIF) [file pone.0117413.s003.tif]
