# Supplementary material for: Analysis of the Link between the Redox State and Enzymatic Activity of the HtrA (DegP) Protein from Escherichia coli
Source: PLoS One. 2015 Feb 24;10(2):e0117413. doi: 10.1371/journal.pone.0117413 (PMC4339722; doi:10.1371/journal.pone.0117413)
Supplement: S1 Table — (DOC) [file pone.0117413.s004.doc]

**Table S1.** Fluorescence lifetime properties of the Trp residues introduced into HtrA.

| **ΔCysW (HtrA-C57A/C69A/F63W/S210A)** | **temperature** | **τ_1_ [ns]** | **f_1_ [%]** | **τ_2_ [ns]** | **f_2_ [%]** | **τ_3_ [ns]** | **f_3_ [%]** | **τ_0_ [ns]** |
| --- | --- | --- | --- | --- | --- | --- | --- | --- |
|  | 20°C | 3.15 ± 0.08 | 48.34 | 0.79 ± 0.05 | 9.96 | 6.92 ± 0.08 | 41.7 | **4.49** |
|  | 25°C | 3.00 ± 0.08 | 49.62 | 0.78 ± 0.05 | 10.82 | 6.68 ± 0.07 | 39.56 | **4.22** |
|  | 30°C | 2.80 ± 0.08 | 51.17 | 0.70 ± 0.04 | 11.55 | 6.41 ± 0.09 | 37.28 | **3.91** |
|  | 35°C | 2.68 ± 0.07 | 52.89 | 0.72 ± 0.04 | 13.6 | 6.22 ± 0.08 | 33.52 | **3.60** |
|  | 40°C | 2.51 ± 0.06 | 54.41 | 0.69 ± 0.04 | 15.17 | 5.97 ± 0.09 | 30.43 | **3.29** |
|  | 45°C | 2.29 ± 0.07 | 55.59 | 0.66 ± 0.04 | 16.94 | 5.68 ± 0.11 | 27.47 | **2.94** |
| **oxidized control**  **(HtrA-F63W/S210A)** | **temperature** | **τ_1_ [ns]** | **f_1_ [%]** | **τ_2_ [ns]** | **f_2_ [%]** | **τ_3_ [ns]** | **f_3_ [%]** | **τ_0_ [ns]** |
|  | 20°C | 2.27 ± 0.09 | 46.41 | 0.52 ± 0.05 | 14.01 | 5.53 ± 0.08 | 39.57 | **3.32** |
|  | 25°C | 2.20 ± 0.08 | 47.55 | 0.52 ± 0.04 | 16.19 | 5.35 ± 0.09 | 36.26 | **3.07** |
|  | 30°C | 2.02 ± 0.09 | 48.4 | 0.46 ± 0.04 | 17.34 | 5.12 ± 0.09 | 34.26 | **2.81** |
|  | 35°C | 1.99 ± 0.09 | 50.17 | 0.46 ± 0.04 | 19.94 | 5.12 ± 0.12 | 29.9 | **2.62** |
|  | 40°C | 1.84 ± 0.07 | 50.43 | 0.41 ± 0.03 | 21.19 | 4.89 ± 0.10 | 28.37 | **2.40** |
|  | 45°C | 1.90 ± 0.08 | 53.3 | 0.43 ± 0.03 | 24.96 | 5.29 ± 0.16 | 21.74 | **2.27** |

τ*_i_*, individual fluorescence lifetime of component *i*; f*_i_*, fractional amount of component *i*; τ_0_, mean fluorescence lifetime.
